# Functions of BLM Helicase in Cells: Is It Acting Like a Double-Edged Sword?

**DOI:** 10.3389/fgene.2021.634789

**Published:** 2021-03-12

**Authors:** Ekjot Kaur, Ritu Agrawal, Sagar Sengupta

**Affiliations:** Signal Transduction Laboratory-2, National Institute of Immunology, New Delhi, India

**Keywords:** BLM helicase, tumor suppressor, oncogene, RecQ helicase, neoplastic transformation

## Abstract

DNA damage repair response is an important biological process involved in maintaining the fidelity of the genome in eukaryotes and prokaryotes. Several proteins that play a key role in this process have been identified. Alterations in these key proteins have been linked to different diseases including cancer. BLM is a 3′−5′ ATP-dependent RecQ DNA helicase that is one of the most essential genome stabilizers involved in the regulation of DNA replication, recombination, and both homologous and non-homologous pathways of double-strand break repair. BLM structure and functions are known to be conserved across many species like yeast, *Drosophila*, mouse, and human. Genetic mutations in the BLM gene cause a rare, autosomal recessive disorder, Bloom syndrome (BS). BS is a monogenic disease characterized by genomic instability, premature aging, predisposition to cancer, immunodeficiency, and pulmonary diseases. Hence, these characteristics point toward BLM being a tumor suppressor. However, in addition to mutations, *BLM* gene undergoes various types of alterations including increase in the copy number, transcript, and protein levels in multiple types of cancers. These results, along with the fact that the lack of wild-type BLM in these cancers has been associated with increased sensitivity to chemotherapeutic drugs, indicate that BLM also has a pro-oncogenic function. While a plethora of studies have reported the effect of *BLM* gene mutations in various model organisms, there is a dearth in the studies undertaken to investigate the effect of its oncogenic alterations. We propose to rationalize and integrate the dual functions of BLM both as a tumor suppressor and maybe as a proto-oncogene, and enlist the plausible mechanisms of its deregulation in cancers.

## Introduction

Both prokaryotic and eukaryotic genomes continuously accumulate spontaneous and genotoxic agent-induced DNA damages that are generated during the DNA replication process and also when the cells are exposed to multiple types of exogenous factors including exposure to chemicals or ionizing irradiation (IR) (Khanna and Jackson, 2001; Giglia-Mari et al., 2011). DNA repair can be classified as a highly complex biological process that orchestrates to detect and repair these genetic insults. DNA repair processes are evolutionarily conserved across different species, and inability to repair the damage can cause mutations and eventually lead to multiple ailments including neoplastic transformation in mammals. Apart from the DNA repair–cell cycle checkpoints, mechanisms enable the restoration of the damaged DNA by halting the progression of cell cycle (Barnum and O'Connell, 2014). DNA damage response (DDR) is a multistep process involving detection of DNA damage and cell cycle checkpoint activation, along with DNA repair, that is ultimately responsible for the repair of aberrant DNA structures and resolution of DNA replication stalled forks. Thus, DDR ensures the transmission of identical genomes to subsequent progenies and thereby maintains the genomic integrity.

Double-strand breaks (DSBs) are one of the most lethal forms of DNA damage. Two major distinct pathways have evolved in both prokaryotes and eukaryotes for repairing DSBs. These include the homologous recombination repair (HRR) and non-homologous end joining repair (NHEJ) pathways. Twenty-five percent to 50% of the DSBs generated by nucleases in yeast and mammalian cells are repaired by the classical NHEJ (cNHEJ) pathway that occurs in all phases of the cell cycle. However, NHEJ is an error-prone process (Clikeman et al., 2001; Stinson et al., 2020). In contrast to NHEJ, the HHR pathway is potentially error free and is largely restricted to the S phase and G2 phase of the cell cycle (Jasin and Rothstein, 2013). Each one of them independently operates to restore the DNA integrity; however, the mechanism by which the processing of the damaged DNA ends by these two pathways varies.

Many of the key factors in the DSB repair pathways have been identified. Cells lacking these factors have been implicated in various diseases in humans. Several recent reviews have reiterated the role of RecQ helicases as critical regulators of these repair pathways (Newman and Gileadi, 2020; Ahamad et al., 2021; Datta et al., 2021). This review focuses on delineating the functions of one of the RecQ helicase BLM with a particular emphasis on its dual role in cancer.

## RecQ Helicases

DNA helicases are a diverse group of proteins that utilizes the energy from ATP hydrolysis to unwind the duplex DNA, with a few of them involved in displacing other proteins from the DNA, making the template accessible to the replication machinery (Xue et al., 2019; Brosh and Matson, 2020). Due to this function, they are known to be involved in a plethora of cellular processes like bacterial conjugation, DNA replication, repair, recombination, and eukaryotic transcription. Of these, RecQ family of helicases are important members of the superfamily 2 (SF2) helicases, which has been found in bacteria, fungi, animals, and plants (Byrd and Raney, 2012). However, the number of *RecQ* genes vary among different species with one homolog found in *Escherichia coli* and budding yeast (*RecQ* and *Sgs1*, respectively), three members in *Drosophila melanogaster* (*DmBlm, DmRecQL4*, and *DmRecQL5*) (Cox et al., 2019), and seven in *Arabidopsis thaliana* and *Oryza sativa* (Bachrati and Hickson, 2003; Hartung and Puchta, 2006).

Five different *RecQ* genes have been identified in humans (*BLM, WRN, RECQL1, RECQL4*, and *RECQL5*). The proteins encoded by all these genes have a structurally conserved helicase domain containing Walker A and B boxes and a DEAH box that functions in unwinding of the helical structure in an ATP- and Mg^2+^-dependent manner (Bennett and Keck, 2004). Additional domains such as the RQC domain (RecQ C-terminal) and HRDC (Helicase and RNase D C-terminal) are also found in few of the members of RecQ family of proteins (Bennett and Keck, 2004; Guo et al., 2005). In RECQ1-3, protein–protein interactions are mediated by the RQC domain, whereas the HRDC domain present only in RECQ2-3 ensures protein–DNA interactions (Morozov et al., 1997; Liu et al., 1999). In addition to this, a 3′ → 5′ exonuclease domain at the N-terminus of WRN and *Xenopus* FFA-1, a nuclear localization signal at the C-terminal of BLM and WRN (Kaneko et al., 1997; Matsumoto et al., 1997), as well as a mitochondrial localization signal in RECQL4 (De et al., 2012) have also been identified. Different members of the RecQ helicase family are involved in the maintenance of genomic integrity during replication, recombination, and repair in both nucleus (Larsen and Hickson, 2013; Bochman, 2014; Croteau et al., 2014) and mitochondria (De et al., 2012; Gupta et al., 2014). Therefore, mutations in three of the RecQ family members, namely, *BLM, WRN*, and *RECQL4*, lead to Bloom syndrome (BS), Werner syndrome (WS), and Rothmund–Thomson syndrome (RTS), respectively, in humans, whereas in yeast, lack of the Sgs1 induces a hyper-recombination as well as hypersensitivity to a wide range of DNA-damaging agents (Watt et al., 1996). Of the five members in human, this review focuses on the dual role of BLM helicase in the context of cancers.

## BLM Helicase

**Figure 1:**
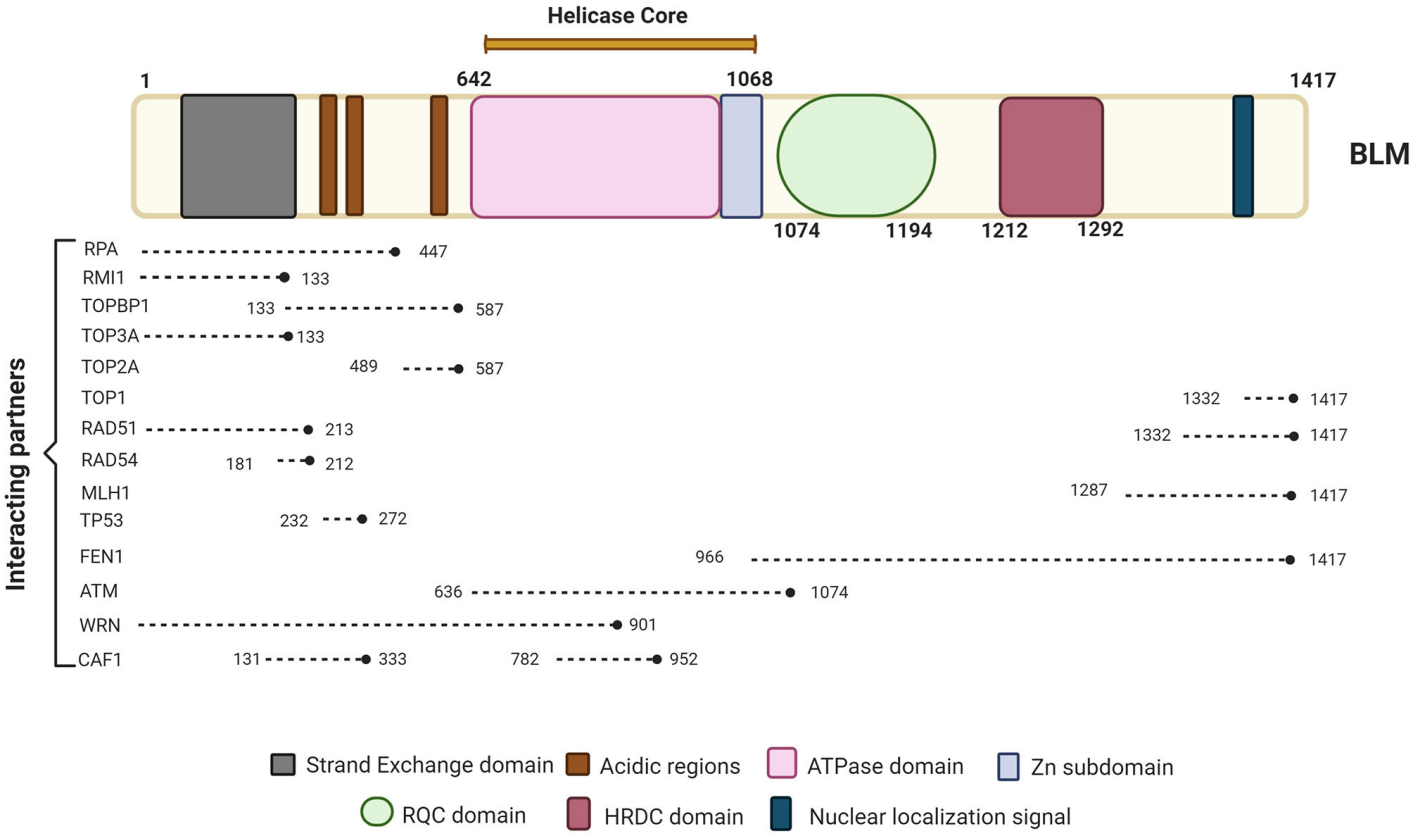
Schematic representation of BLM primary protein structure and its interacting partners. BLM is composed of the structurally ordered ATPase/helicase core, RQC, and HRDC domains in the C-terminal of the protein of which helicase domain is conserved across organisms. Key proteins involved in different repair pathways and interacting with BLM are shown along with their approximate binding positions with respect to BLM. The different interacting partners of BLM that are involved in DNA repair processes and chromatin assemble include RPA (Kang et al., 2018; Bythell-Douglas and Deans, 2021), RMI (Blackford et al., 2015; Bythell-Douglas and Deans, 2021), TOPBP1 (Blackford et al., 2015), TOP3A (Hu et al., 2001; Bythell-Douglas and Deans, 2021), TOP2A (Bhattacharyya et al., 2009), TOP1 (Grierson et al., 2013), RAD51 (Bugreev et al., 2007), RAD54 (Srivastava et al., 2009), MLH1 (Pedrazzi et al., 2001), FEN1 (Sharma et al., 2005), ATM (Beamish et al., 2002), WRN (von Kobbe et al., 2002), and CAF1 (Jiao et al., 2004). Figure created with BioRender.com.

BLM is one of the important members of the RecQ family of DNA helicases. It is a 1417-amino-acid protein-coding gene located on the chromosome 15q26.1, possessing a 3'−5' ATP-dependent helicase activity whose expression is tightly regulated in a cell cycle manner with highest levels observed in late S and G2 phases of the cell cycle (Dutertre et al., 2000; Sengupta et al., 2005). The different domains of BLM interact with a number of proteins—some of which are in a cell cycle-dependent manner (summarized in Figure 1).

## BLM Helicase and Repair Pathways

BLM functions primarily in the DNA replication and repair of DSBs by associating with various HHR factors and replication machinery. BLM associates and forms a BTRR complex or “BLM dissolvasome” consisting of topoisomerase IIIα (TopIIIα) and RecQ-mediated genome instability proteins 1 and 2 (RMI1 and RMI2, respectively) to process the Double Holliday Junctions (dHJs) generated during the strand invasion step of the HRR pathway yielding non-crossover recombinants (Hu et al., 2001; Daley et al., 2014; Bythell-Douglas and Deans, 2021). Notably, the interaction between BLM and Topo IIIα is evolutionary conserved—it occurs in yeast (Gangloff et al., 1994), *E. coli* (Harmon et al., 1999), as well as in somatic and meiotic human cells (Johnson et al., 2000; Wu et al., 2000). In the anaphase population of human cells, this interaction at the ultrafine bridges (UFBs) ensures complete sister chromatid decatenation (Chan et al., 2007). BLM preferentially unwinds multiple types of complex DNA structures including G-quartet, D-loop, telomere DNA, and Holliday Junctions (HJs) (Vindigni and Hickson, 2009). It has been reported that BLM possesses a low helicase activity; however, physical interaction with Replication Protein A (RPA) accentuates its unwinding activity on both intact and nicked ssDNAs (Brosh et al., 2000; Kang et al., 2018; Qin et al., 2020). Recent study identified three conserved RPA binding motifs in the BTRR complex (two in BLM and one in RMI1) that interact with the RPA1 N-terminal OB-fold (Shorrocks et al., 2021). This interaction was found to be specifically required in the role of the BTR complex in promoting replication fork restart but not in its roles of suppressing sister chromatid exchanges (SCEs), processing UFBs, or promoting DNA-end resection (Shorrocks et al., 2021). Furthermore, a critical interaction of BLM with RAD51 is responsible for homology search and during the subsequent strand invasion step (Wang et al., 2000; Wu et al., 2001). BLM also promotes DNA end resection by the exonucleases EXO1 and DNA2, generating a 3′ single-stranded substrate for RAD51 recruitment and filament formation (Mimitou and Symington, 2009; Nimonkar et al., 2011). Together, these properties of BLM position it as a pro-recombinogenic protein. Multiple studies have also shown that BLM accumulates at the stalled replication forks, interacting with FANCM and FANCC to dissolve the dHJs and related DNA configurations (Davalos and Campisi, 2003; Wu and Hickson, 2003; Sengupta et al., 2004; Singh et al., 2008; Moder et al., 2017). Further, we have earlier elucidated that the ATR-mediated phosphorylation at Thr99 on BLM is required for its interaction with the signal transducer 53BP1 protein. This interaction is critical for the anti-recombinogenic role of BLM during the HHR pathway and ensures survival post-replicative stress (Tripathi et al., 2007, 2008), thereby providing a hint about the dual roles of this helicase.

Genome-wide guanine-quadruplex (G4) motif analysis has shown that unconventional structures are particularly enriched in telomeres, minisatellites, ribosomal DNA, and, importantly, gene regulatory regions (Drosopoulos et al., 2015). BLM has been shown to bind and unwind G4 structures promoting fork progression through G-rich telomeric DNA (Drosopoulos et al., 2015; Tippana et al., 2016). BLM has also been implicated in repairing the secondary DNA structures including R-loops and G4s induced by reactive oxygen species (ROS) at transcriptionally active sites (Tan et al., 2020). These studies again provide evidence that BLM suppresses recombination at these telomeric sites to maintain genomic stability (Root et al., 2016; van Wietmarschen et al., 2018).

BLM has also been identified as an early sensor to multiple types of DNA damage (Sengupta et al., 2004; Tripathi et al., 2018). BLM is reported to assemble along with hRAD51 and p53 immediately to the sites of stalled replication (Sengupta et al., 2003; Ouyang et al., 2009) and IR-induced DSBs (Wu et al., 2001). In asynchronously growing cells, Chk1-mediated Ser646 phosphorylation (Kaur et al., 2010) on BLM causes it to colocalize with the promyelocytic leukemia (PML) protein (Bischof et al., 2001). Notably, in response to laser-induced DSBs, BLM co-localizes with γH2AX and ATM within seconds of induction at the sites of damage (Karmakar et al., 2006). The localization of BLM onto the stalled replication forks occurs after its ubiquitylation at lysine residues 105, 225, and 259 by RNF8/RNF168 E3 ligases (Tikoo et al., 2013). The early recruitment of BLM is ATR- and ATM-dependent, and this ensures the optimum formation of pATM and 53BP1 foci during replication stress (Davies et al., 2004). BLM along with BRCA1 and the MRN complex is part of a large complex called BRCA1-associated genome surveillance complex (BASC), which is co-recruited with PCNA during DNA replication-associated repair (Wang et al., 2000). In contrast, BLM recruitment in the later stages of repair is independent of ATM but requires functional interaction between polyubiquitylated BLM and NBS1 for its retention at the DSB site (Tripathi et al., 2018). In addition, BLM has been shown to physically and functionally associated with hp150, the largest subunit of chromatin assembly factor 1 (CAF-1) to promote survival in response to DNA damage and/or replication blockade (Jiao et al., 2004). Furthermore, a functional interaction between BLM and RAD54 enhances the chromatin remodeling activity of RAD54 resulting in its increased recruitment of RAD51 protein onto the HU-induced DNA damage (Srivastava et al., 2009). These results indicate that the role of BLM in the DDR response is a combination of its role as an early DNA damage sensor as well as its multiple functions during the effector stage of the repair.

Recently, we have also demonstrated that BLM is co-recruited with the c-NHEJ factor XRCC4 in a cell cycle-specific manner and regulates the cNHEJ process (Tripathi et al., 2018). In a cell cycle phase-dependent manner, BLM seems to help in making the choice between HR and cNHEJ (Tripathi et al., 2018). Apart from its role in HHR, BLM also has an effect on the other DNA repair pathways operative in human cells. BLM prevents the activation of the error-prone MMEJ pathway in human and mouse. Thus, cells lacking BLM displayed higher genomic rearrangements (Gaymes et al., 2002). In addition, studies have revealed that the C-terminal of human BLM interacts with the mismatch repair protein MLH1; however, this interaction did not seem to affect the post-replicative mismatch repair pathway (Langland et al., 2001; Pedrazzi et al., 2001).

## Regulation of BLM

BLM has been shown to undergo various post-translational modifications (PTMs) including phosphorylation, ubiquitination, acetylation, and SUMOylation that are necessary for its function, interaction, turnover, localization, and stability. In turn, these PTMs of BLM have been shown to regulate several DDR signaling cascades. BLM undergoes phosphorylation at Thr99 and Thr122 by ATM/ATR that is crucial for restarting of stalled replication folks after HU or IR treatment (Davies et al., 2004). Constitutive phosphorylation of BLM at Serine 502 by Chk1 during interphase stabilizes its levels, preventing its cullin-3-mediated degradation in colon cancer cells (Petsalaki et al., 2014). Additionally, NEK11-dependent S phase-specific phosphorylation at Serine 338 of BLM mediates its interaction with TopBP1 that functions to stabilize the BLM levels in S and G2 phases of the cell cycle (Wang et al., 2013).

Apart from phosphorylation, K63-linked ubiquitination of BLM at Lys105, Lys225, and Lys259, mediated by RNF8/RNF168, was demonstrated to be essential for BLM to relocate to the sites of stalled replication (Tikoo et al., 2013). K48-linked ubiquitylation of BLM by E3 ligase, Fbw7α, leads to its subsequent degradation during mitosis. This modification, in turn, is regulated by sequential phosphorylation on BLM by multiple kinases at Thr182, Thr171, and Ser175 residues (Kharat et al., 2016). Further, K3 linked BLM ubiquitination by MIB1 E3 ligase that led to its rapid degradation in the G1 phase of the cell cycle (Wang et al., 2013). PTM-like SUMOylation of BLM at Lys317, Lys331, Lys34, and Lys347 is also shown to be necessary for the interaction between BLM and RAD51 promoting HR repair (Eladad et al., 2005; Ouyang et al., 2013).

**Figure 2:**
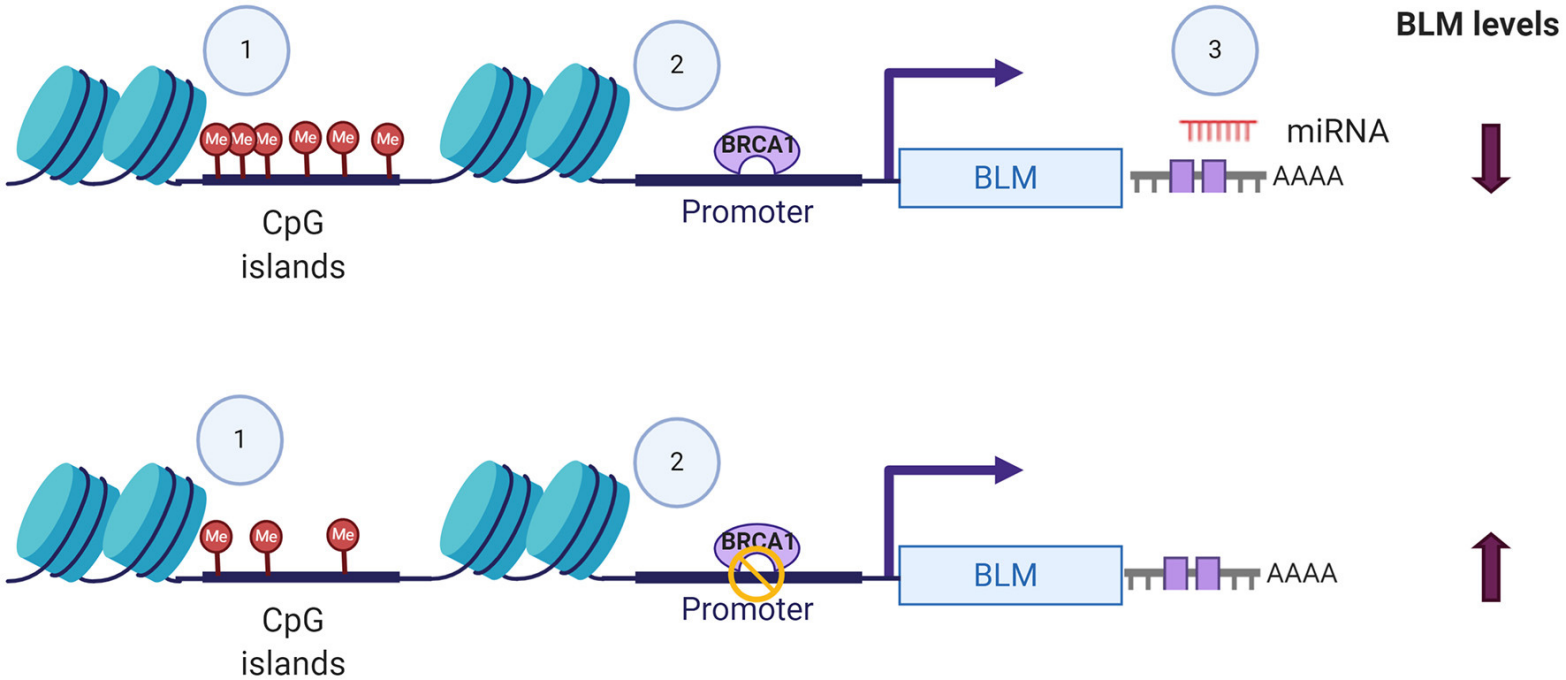
Different modes of BLM transcript regulation. The alterations in the expression levels of BLM can be brought about by (1) CpG island methylation, (2) transcriptional regulators like BRCA, and (3) miRNA-mediated targeting of 3′-UTR. Figure created with BioRender.com.

In addition to its post-translational regulation, recent evidences of its post-transcriptional regulation particularly in cancers have also been demonstrated. miR-522-3p was found to be highly expressed in colorectal cancer (CRC) tissues compared to adjacent non-tumor tissues and negatively regulates the BLM levels, promoting proliferation of colon cancer cells and thus demonstrating its tumor suppressor function (Shuai et al., 2018). In contrast, overexpression of miR-607 and miR-27b-3p in PC3 cells reduced proliferation, colony formation, and invasion capacity by decreasing the BLM mRNA levels and protein levels, respectively (Figure 2) (Chen Y. et al., 2019). BLM transcript levels have also been found to undergo epigenetic regulation by CpG island promoter methylation in CRC samples (Votino et al., 2017). CpG island promoter hypomethylation altered its expression, which might contribute to proliferation of poorly differentiated cells (Votino et al., 2017). The different modes by which BLM transcript levels can be regulated are summarized in Figure 2.

## Clinical Manifestations Due to Alterations in BLM Levels

BS is caused by either homozygous or compound heterozygous mutations in the *BLM* gene located at the 15q26.1 locus (Ellis et al., 1995a; German et al., 2007). BS patients display features like proportional pre- and postnatal dwarfism, immunodeficiency, hypersensitivity to sunlight, infertility in males, subfertility in females, and type 2 diabetes mellitus (Bloom, 1954; German and Passarge, 1989; Ellis et al., 2008). Due to its pivotal role as an anti-recombinogenic protein, BS patients lacking the functional protein exhibit a significant increase in mitotic recombination, high rates of heterozygosity (Langlois et al., 1989), chromatid gaps, breaks, increased frequency of SCE (Chaganti et al., 1974; German et al., 1974; German, 1993; Ellis et al., 1995b), telomere defects (TD) (Barefield and Karlseder, 2012), and aberrant quadriradial chromosomes (Chaganti et al., 1974; Lonn et al., 1990; Groden and German, 1992). Additionally, BLM defective cells displayed accumulation of anaphase bridges that caused chromosome entanglement (Chan et al., 2007). Since the cells cannot adequately repair the inherent and induced DNA damage, BS patients additionally show increased sensitivity toward DNA-damaging agents like HU, camptothecin (CPT), and IR (Davies et al., 2004; Ouyang et al., 2008; Shastri and Schmidt, 2016).

Most of the BS mutations are either non-sense or frameshift mutations causing premature truncation of the protein. Additionally, several of the missense mutations spreading across the helicase domain and RQC domain have also been identified and reported in the Bloom Syndrome Registry and BLM database (Ellis et al., 1995a; German et al., 2007; Bythell-Douglas and Deans, 2021). These missense mutations have been shown to abolish the ATPase and DNA binding activity with some of them losing ATP binding activity, thus rendering the BLM protein catalytically inactive (Bahr et al., 1998; Rong et al., 2000; Guo et al., 2007). The Ashkenazi Jewish are the most commonly affected population by BS because of the high prevalence of the BLM^Ash^ founder mutation: a 6-bp deletion and 7-bp insertion at the nucleotide position 2281 in BLM cDNA (Li et al., 1998). However, the BLM^Ash^ mutation has also been found in non-Jewish individuals, such as Americans of Spanish descent (Ellis et al., 1998).

Similar to BS patients, individuals harboring mutations in the *TOP3A* (an essential gene in mammals) displayed elevated rates of SCE, unresolved recombination, and replication intermediates, leading to chromosome bridges and thus inducing genomic instability (Martin et al., 2018). Additionally, homozygous truncating variants in RMI1, another important member of the BTR complex, caused growth retardation as seen in the case of BS (Martin et al., 2018). These reports establish that the intact, functionally active BTRR complex is required for the regulation of recombination repair and thus in genome maintenance.

BLM is also postulated to be involved in the development and maintenance of the immune system. In BS patients, abnormal serum concentrations of at least one subclass of serum immunoglobulins with IgM and IgA levels and lowered IgG levels have been documented (Hutteroth et al., 1975; Weemaes et al., 1979; Taniguchi et al., 1982; Kondo et al., 1992). Upon BLM depletion, the number of progenitor B lymphoid cells in the bone marrow and mature B cells in the spleen and peritoneal cavity was significantly decreased in the B cell-specific BLM knockout mice (Babbe et al., 2009). Additionally, ablation of BLM in mice and in BS patients also leads to defect in the T cell lineage (Hutteroth et al., 1975; Taniguchi et al., 1982; Van Kerckhove et al., 1988). It was observed that in some BS patients, the reduced CD4-positive T cell numbers (Van Kerckhove et al., 1988) impaired T cell proliferation, and T helper function has been identified (Hutteroth et al., 1975; Taniguchi et al., 1982; Van Kerckhove et al., 1988). Using the conditional T-cell-specific BLM knockout mice, severe blockage at the β selection checkpoint was observed, which resulted in significantly decreased number of thymocytes (Babbe et al., 2007). Due to an accumulation of damaged DNA and micronuclei in BLM-deficient cells, enhanced expression of *inflammatory interferon-stimulated gene (ISG)* and increased levels in peripheral blood have also been observed. This increased expression is mediated through the Cyclic GMP-AMP synthase–stimulator of interferon genes–interferon regulatory factor-3 (cGAS–STING–IRF3) cytosolic DNA–sensing pathway, thus linking the innate immune system with the DNA damage machinery (Gratia et al., 2019). However, the specific molecular mechanisms regulated by BLM are not well-elucidated. Altogether, BLM plays an indispensable role in the development, proliferation, maintenance, stability, and function of immune cells and contributes to the immune deficiency in patients afflicted with BS.

## Tumor-Suppressive Functions of BLM

In addition to the aforementioned clinical features, loss of functional BLM increases the risk of developing plethora of solid tumors and hematological malignancies in BS patients (German et al., 2007; Cunniff et al., 2017). BLM-depleted cells lead to amassment of damaged DNA, showed suppressed cell proliferation, and enhanced genomic damage with high response or sensitivity toward various chemotherapeutic drugs like cis-diamminedichloroplatinum (CDDP or cis-Pt), CPT, HU (Arora et al.), and 5-fluorouracil (5-FU) (Mao et al., 2010), suggesting its tumor-suppressive function. Studies using mouse models further demonstrated that haploinsufficiency of BLM led to an early onset of lymphomas and intestinal tumors particularly CRC (Gruber et al., 2002; de Voer et al., 2015). BLM heterozygous mutant mice developed T cell lymphoma at a much more rapid rate when challenged with murine leukemia virus (Goss et al., 2002) and the frequency of intestinal tumor development is higher when crossed with *Adenomatous Polyposis Coli (APC)* gene heterozygous mutant mice. In contrast, BLM transgenic mice expressing human BLM attenuated intestinal tumors when crossed with APC heterozygous mutant mice, thereby indicating that tumor growth can be regulated in a BLM dose-dependent manner (McIlhatton et al., 2015). BLM homozygous null BLM^m3/m3^ mice are viable, fertile, and more cancer prone with and without tumor predisposing factors like gamma irradiation (Warren et al., 2010). This tumorigenic effect was enhanced after irradiation of BLM^m3/m3^ mice (Warren et al., 2010) wherein lymphoma, sarcoma, and carcinoma were the most common cancers arising in this genotype. It is important to note that the hematopoietic system is predominantly affected by the lack of wild-type BLM expression as the frequencies of lymphoma and leukemia in BS are higher than expected, the most common being the T cell lymphoma (Luo et al., 2000; Warren et al., 2010). In patched homolog 1 (Ptch1) heterozygous mutant mice, loss of BLM function significantly enhanced the tumorigenesis of basal cell carcinoma (BCC) (a type of skin cancer) and rhabdomyosarcomas (RMS) (Davari et al., 2010). Conditional BLM knockout mice bearing heat shock promoter cre transgene (HS-cre), prostate-specific antigen promoter-cre transgene (PSA-cre), and ovine beta-lactoglobulin promoter-cre transgene (BLG-cre) develop different types of mammary tumors, i.e., adeno-myoepithelioma and adenocarcinoma (Chester et al., 2006). Cell lines developed from these mammary tumors produce a high number of SCEs and show high chromosomal instability (CIN) (Chester et al., 2006). Thus, in mice, BLM acts as a factor essential for maintaining genomic stability and is involved in the prevention or reduction of tumor development (McDaniel et al., 2003).

Similar to mice models, BS patients also develop a spectrum of cancers at a very early age, of which leukemia and lymphomas are the most common malignancies followed by CRCs (German et al., 2007; Cunniff et al., 2017). This was particularly observed in the Ashkenazi Jews population harboring heterozygous BLM mutation, which displayed a more than a 2-fold increase in colon cancer incidence (Li et al., 1998). Furthermore, hematological malignancies of BS patients have been reported to demonstrate chromosomal rearrangements (Kaneko et al., 1996; Schuetz et al., 2009). Elevated incidence of micronuclei in the exfoliated epithelial cells from the BS patients compared to normal individuals carrying a heterozygous *BLM* gene mutation has also been reported (Rosin and German, 1985), indicative of HHR deregulation and the presence of genomic instability. While investigating its function in during instability in CRC, BLM deficiency was found to induce hyper-recombination in epithelial cells that was associated with loss of heterozygosity (Traverso et al., 2003).

In accordance with its role as a caretaker of the genome, several of the germline mutations in the *BLM* gene have been identified to be associated with CRC risk (Sokolenko et al., 2012; de Voer et al., 2015). The whole exome sequencing data from the CRC patients was used to infer that about 0.11% of the general population were enriched with the heterozygous BLM mutation that confers low-to-moderate penetrance risk for developing CRC. The carrier frequency of this mutation was, however, observed to be higher by about 1% in the people with an Ashkenazi Jewish ancestry (de Voer et al., 2015). Gruber et al. similarly identified that CRC patients were high-frequency carriers of the heterozygous BLM^Ash^ mutation (Gruber et al., 2002). However, in the case of the association of BLM mutation with breast cancer, a contradictory set of reports exists in the literature. While Sokolenko et al. and Prokofyeva et al. identified that truncating mutation of BLM (c.1642 C>T, p.Gln548Ter) conferred a 6-fold increased risk of breast cancer, such association was not observed by Kluzniak et al. in the large cohort of samples obtained from Poland (Sokolenko et al., 2012; Prokofyeva et al., 2013; Kluzniak et al., 2019). A similar observation with the BLM^Ash^ and p.Gln548Ter mutations was observed in the prostate cancer (PC) cells wherein no significant effect on the survival was seen even though the frequency of truncating BLM germline mutations was higher in advanced PC patients as compared to the control populations (Antczak et al., 2013; Bononi et al., 2020; Ledet et al., 2020). Based on these observations, it was hypothesized that the presence of only one functional allele of BLM is incapable of maintaining genomic integrity, which could lead to accumulation of high frequency of deleterious mutations in the cell harboring BLM mutation. In addition, the occurrence of such mutations in the colonic cancer stem cells could potentially generate a hyper-mutated cancer phenotype (Gruber et al., 2002).

Mechanistically, it was demonstrated that in colon cancer cells, BLM enhanced Fbw7α-mediated K48-linked ubiquitylation of proto-oncogene c-Myc (Chandra et al., 2013). This subsequently led to enhanced c-Myc degradation by proteasomal pathway. Additionally, BLM also alleviated c-Jun degradation by E3 ligase Fbw7α, thus attenuating the proliferation of colon cancer cells in mouse xenograft model (Priyadarshini et al., 2018). Thus, the lack of functional BLM may hamper its ability to regulate the expression of these proto-oncogenes, causing the promotion of tumorigenesis. In addition, BS cells harboring p53 mutations exhibited a lower level of apoptosis and DNA repair and thus may negatively regulate the BLM-dependent repair pathway (Wang et al., 2001). It is noteworthy that BS patients exhibited significant differences in their mRNA expression profile as compared to the normal fibroblasts, with genes involved in cell proliferation and survival being the topmost altered genes (Nguyen et al., 2014; Montenegro et al., 2020).

## Evidence Supporting the Oncogenic Functions of BLM

**Figure 3:**
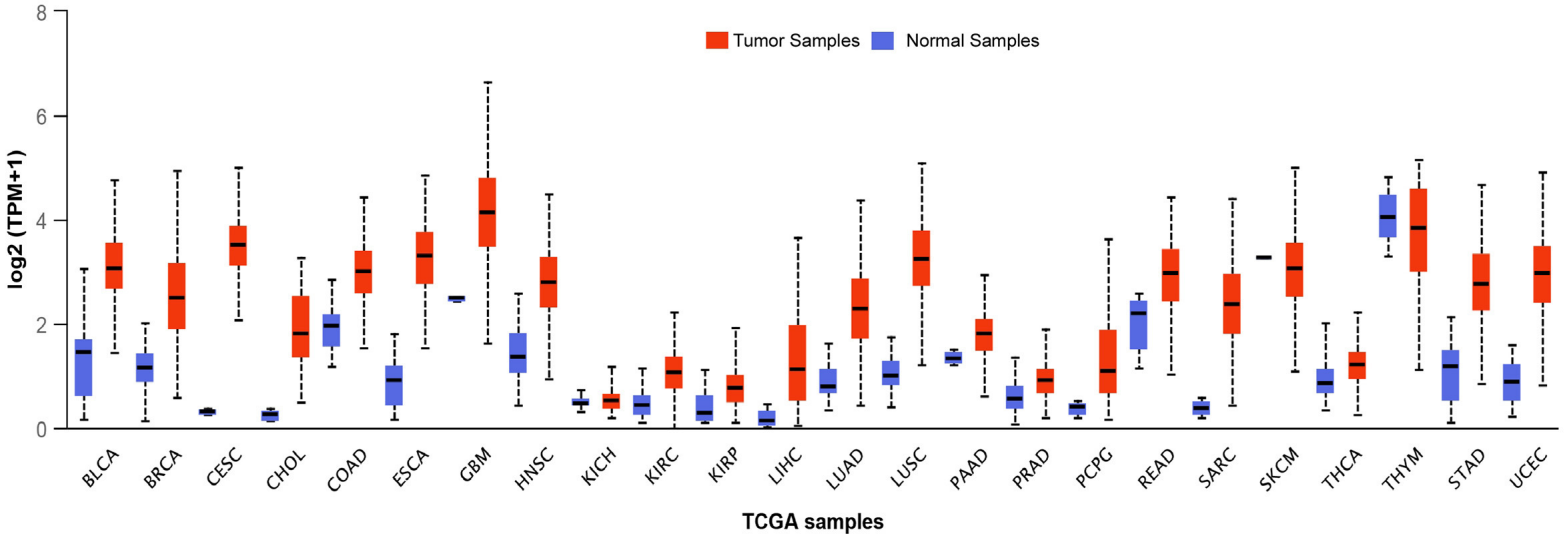
Transcript levels of BLM across different types of tumor samples as well as their matched normal samples. The expression levels of BLM in different tumor samples along with their normal samples as analyzed using the TCGA datasets: BLCA (Urothelial Bladder Carcinoma), Breast Invasive Carcinoma (BRCA), CESC (Cervical Squamous Cell Carcinoma and Endocervical Adenocarcinoma), CHOL (Cholangiocarcinoma), COAD (Colon adenocarcinoma), ESCA (Esophageal carcinoma), GBM (Glioblastoma), HNSC (Head-Neck Squamous Cell Carcinoma), KICH (Kidney Chromophobe), KIRP (Kidney renal papillary cell carcinoma), LHC (Liver hepatocellular carcinoma), LUAD (Lung adenocarcinoma), LUSC (Lung squamous cell carcinoma), PAAD (Pancreatic adenocarcinoma), PCPG (Pheochromocytoma and Paraganglioma), READ (Rectum adenocarcinoma), SARC (Sarcoma), SKCM (Skin Cutaneous Melanoma), THCA (Thyroid Cancer), THYM (Thyroid carcinoma), STAD (Stomach adenocarcinoma), and UCEC (Uterine Corpus Endometrial Carcinoma) (Chandrashekar et al., 2017).

BLM expression levels are found to be high in testis, ovary, hematopoietic cells, and in all the proliferative cells (like cells of lymphoid origin, in the skin, and digestive tract) (Turley et al., 2001). This upregulation of BLM in forebear cells or undifferentiated cells indicates that BLM may be involved in controlling the differentiation of cells as its overexpression has been associated with the suppression of the differentiating markers (Turley et al., 2001). Taking this evidence into consideration, it can be argued that in contrast to tumor suppressor, BLM may be involved in promoting cancer development. In fact, BLM protein expression was observed in the tumors of both lymphoid and epithelial origin wherein a significant correlation between Ki67 and PCNA was observed with BLM expression (Chandrashekar et al., 2017). Furthermore, an *in silico* examination of the TCGA datasets revealed that BLM mRNA is overexpressed in all types of cancer tissues as compared with normal tissues (Figure 3) (Chandrashekar et al., 2017). A recent report utilizing the computation approach has also identified that BLM overexpression was related to poor overall survival (OS) in lung and gastric cancer patients and thus may act as a critical prognostic marker for the detection of these cancers (Alzahrani et al., 2020). In particular, exceedingly high levels of BLM have been demonstrated in all of the hematological malignancies such as intense myeloid leukemia, constant lymphocytic leukemia, lymphoma, and different myeloma (Turley et al., 2001). In acute myeloid leukemia (AML) samples with normal karyotype, high expression of BLM displayed a strong association with poor prognosis, whereas with abnormal karyotype, high expression of BLM associated with better OS (Viziteu et al., 2016a). Further, it was observed that BCR/ABL tyrosine kinase (a common tyrosine kinase fusion in chronic myeloid leukemia) as well as other fusion tyrosine kinases induced the expression of BLM and its helicase function (Slupianek et al., 2005). This in turn potentiated HHR repair capacity via its interaction with RAD51 complex for HR repair in response to chemotherapeutic drugs including cisplatin and mitomycin C, thus playing a role in BCR/ABL-induced resistance to these genotoxic insults (Slupianek et al., 2005).

High levels of BLM protein were also observed in PC cell lines and patients, exhibiting an enhanced rate of cell proliferation whereas BLM depletion resulted in an increased rate of apoptosis due to enhanced ROS generation mediated through the inhibition of AKT and PRAS40 signaling (Chen K. et al., 2019). Thus, BLM may induce oncogenesis through activating pAKT and pPRAS40 in PC (Chen K. et al., 2019). BRCA1 is one of the transcriptional regulators known to be involved in PC (De Luca et al., 2011, 2013). BRCA1 expression hindered tumor development and sensitized these cells to chemotherapy, whereas its suppression promoted chemoresistance. BLM is negatively regulated by BRCA1. Hence, upon BRCA1 suppression, BLM level is elevated, which conceivably leads to chemoresistance upon DNA damage (De Luca et al., 2011; Qian et al., 2017).

Further, BLM mRNA and protein levels are found to be overexpressed in CRC cell lines and patients (Lao et al., 2013). A meta-analysis of the gene expression data sets revealed that BLM levels were significantly upregulated in a subset of poorly differentiated CRC samples wherein shorter relapse-free survival was seen (Votino et al., 2017). In these CRC samples, a positive correlation between BLM expression levels and molecular parameters of the tumors like CpG island methylator phenotype (CIMP) and DNA mismatch repair was observed (Votino et al., 2017). Notably, aberrant overexpression of BLM has been reported to lead to its mis-localization to the cytosol instead of the nucleus and thereby compromising its DNA repair activity (Votino et al., 2017) in CRC cells. CRC samples with low BLM mRNA levels were found to be sensitized with the mitomycin C treatment, thereby showing better survival; in contrast, resistant CRC cell lines had elevated BLM levels (Kwakman et al., 2015).

A study conducted on about 2000 breast tumor samples also revealed that BLM mRNA overexpression was significantly associated with high histologic grade, larger tumor size, estrogen receptor, and progesterone receptor status (Arora et al., 2015). Furthermore, a significant BLM mRNA overexpression along with high BLM cytoplasmic localization was observed in the aggressive molecular phenotypes (including PAM50, which is a 50-gene signature that classifies breast cancer into five molecular intrinsic subtypes: Luminal A, Luminal B, HER2-enriched, Basal-like, and Normal-like) and has been associated with poor breast cancer-specific survival, possibly highlighting BLM transcript level detection as a promising biomarker (Arora et al., 2015).

Re-expression of reverse transcriptase telomerase (Shay and Bacchetti, 1997) or alternative lengthening of telomeres (ALT) (Bryan et al., 1997) have been implicated in the acquisition of replicative immortality by cancer cells. These ALT positive cancer cells display a highly complex karyotype with excessively clustered telomeres localized in specialized PML nuclear bodies called ALT-associated PML bodies (APBs) (Yeager et al., 1999; Draskovic et al., 2009). It is at these sites where ALT-dependent telomere recombination has been shown to occur. BLM has been shown to contribute in telomere maintenance through its capacity of dissolution and alleviating late-replicating structures (LRI) (Barefield and Karlseder, 2012). Using biophysical studies, Min et al. established that BLM helicase activity is vital for the generation of single-stranded telomeric DNAs and accumulation of RPA at telomere clustering scaffolds (Min et al., 2019). Notably, through its interaction with the shelterin protein Telomeric Repeat Binding Factor 2 (TRF2), BLM facilitates efficient telomere extension (Lillard-Wetherell et al., 2004) that is dependent on the PML-mediated localization of the BTR complex in ALT cells (Loe et al., 2020). On the other hand, its association with Telomeric Repeat Factor 1 (TRF1) inhibits BLM unwinding activity of telomeric substrates (Lillard-Wetherell et al., 2004). Further, SLX4 interacting protein (SLX4IP), FANCM, and FANCD2 have been identified as critical regulators of ALT phenotype, limiting the deregulated activity of BLM (BTR complex) on the telomeres, thus ensuring appropriate balance of its resolution activities at the recombining telomeres (Root et al., 2016; Panier et al., 2019; Silva et al., 2019). Loss or inactivation of these regulators may promote growth of ATL cancer cells in a BLM-dependent manner.

Based on the importance of the helicase-dependent function of BLM, a selective small-molecule inhibitor, ML-216, was synthesized and was demonstrated to reduce proliferation and increase SCE in cellular studies on human cultured cells (Rosenthal et al., 2010; Nguyen et al., 2013). The inhibitor has also shown promise in inducing a significant amount of apoptosis in patient-derived primary myeloma cells having aberrant BLM expression as compared to normal bone marrow (Viziteu et al., 2016b). Based on these observations, a class of Isaindigotone derivatives has been found as a novel BLM inhibitor, attenuating DNA damage-dependent recruitment of BLM, thus affecting the HRR process (Yin et al., 2019). In addition, evaluation of quinazolinone derivatives led to the identification of another BLM helicase inhibitor that has shown promise in sensitizing the CRC cell in combination with chemotherapy drugs and PARP inhibitors (Wang et al., 2020). However, these inhibitors may lack specificity requiring further refinement before they can be used as anticancer agents.

## Perspective

**Figure 4:**
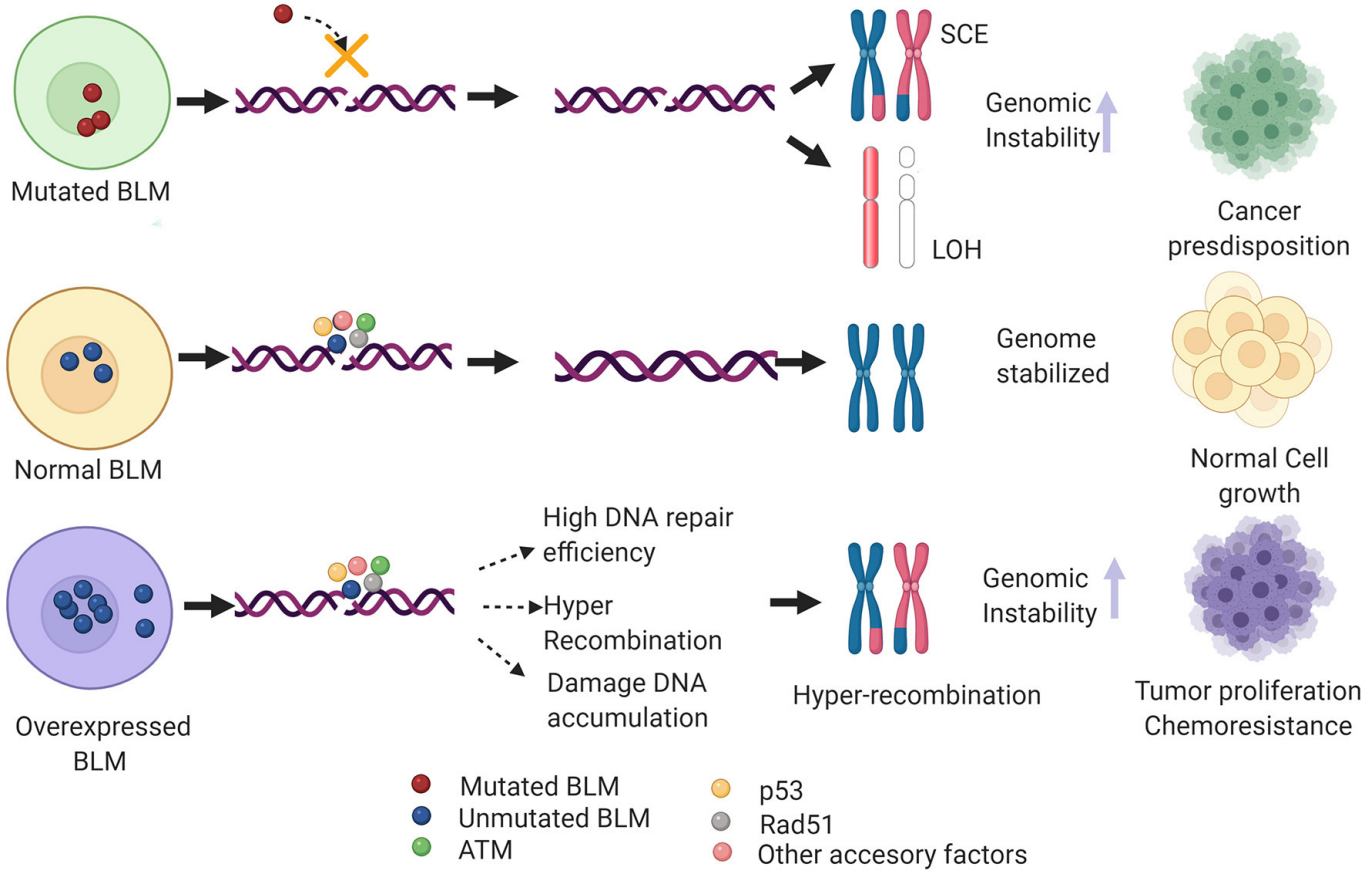
Hypothetical model depicting the clinical phenotype of de-regulated BLM levels. In case of mutant *BLM* (as seen in BS patients), inefficient DNA repair via HRR induces formation of sister chromatid exchanges (SCEs) and loss of heterozygosity (LOH), and in turn alleviates genomic instability and thus predisposing to multiple types of cancers. On the other hand, overexpressed BLM mislocalizes to cytoplasm that may heighten the DNA repair capacity and cause hyper-recombination, thus promoting tumor growth and chemoresistance. Additionally, BLM overexpression may dislocate the RAD51 filaments, preventing efficient HRR pathway and eventually causing accumulation of DNA damage. Figure created with BioRender.com.

Nearly 100 years since the identification of the first DNA repair pathway, extensive research in this field has led to the identification of repair factors critical for the survival/fitness of both prokaryotes and eukaryotes. Thus, genetic ablation of these has been associated with various diseases in humans particularly cancer. Notably, several of these critical regulators of repair have been identified as a potential therapeutic target for cancers as well as other genetic abnormalities associated with DDR factors. One such example is that of the early response gene ATM whose role in mediating cancer resistance has been well-elucidated and thus several of the ATM inhibitors are under different phases of the clinical trials (Lavin and Yeo, 2020). BLM helicase has been implicated in various DNA transactions where it acts as a bonafide tumor suppressor gene. However, recent evidence shows BLM mRNA to be overexpressed in a plethora of cancers including colon, breast, and hematological cancers when compared with the normal samples (Alzahrani et al., 2020). Additionally, it has been postulated that non-sense SNP-mediated aberrant BLM activity or its high mRNA expression levels could confer genomic instability in humans, predisposing them to different cancer types (Alzahrani et al., 2020). From the above studies, it can be extrapolated that an optimal level of BLM is necessary to maintain genome stability. Both high and low levels or loss of BLM may lead to genomic instability and may eventually promote tumorigenesis (Figure 4).

These observations have provoked the question as to whether BLM helicase performs dual functions in different types of cancer. Perhaps a more pertinent query will also be—when does BLM act as a tumor suppressor and when does it convert into a proto-oncogene? In order to address these questions, it becomes imperative to investigate the molecular mechanisms involved in the de-regulation of the *BLM* gene specifically in the cancer cells to gain insights into its “dual role” in humans. The role of PTMs of BLM has been well-elucidated (Bohm and Bernstein, 2014). Ubiquitylation at specific residues of BLM has been found to regulate its stability in a cell cycle-specific manner (Wang et al., 2013; Kharat et al., 2016). However, the status of these PTMs as well as their effect on BLM turnover in the context of cancer progression has not yet been examined. It is also possible that a single or combination of yet undiscovered PTM on BLM acts like a trigger that converts BLM from a tumor suppressor into an oncogene. Recent evidence has also shed light on how BLM undergoes miRNA-mediated post-transcriptional control (Shuai et al., 2018; Chen Y. et al., 2019). It is possible that miRNA-mediated BLM turnover can be altered in specific types of cancers. In addition to this, there has been a growing interest in elucidating the importance of epigenetic regulators in BLM expression in cancers. A recent report identified that hypomethylation of BLM promoter at the CpG islands enhanced the BLM expression in colon cancer cells (Votino et al., 2017). This resulted in high levels of BLM expression, which mis-localized to the cytoplasm due to which a heightened DDR was seen in the tumor samples (Votino et al., 2017). Interaction studies have shown that BLM has functional interactions with two of the chromatin modifiers CAF1 and RAD54 during DDR (Jiao et al., 2004; Srivastava et al., 2009). Whether these interactions also have an effect on the BLM function in cancer needs to be explored further. Deregulation of tumor suppressor and the high risk of cancer development have been well-documented (Sherr, 2004; Giancotti, 2014). Many of these tumor suppressors also function as transcriptional regulators and may inter-regulate each other in a coordinated or backhanded way (el-Deiry et al., 1993; Liu et al., 2008; De Luca et al., 2011). A few of the major tumor suppressors like BRCA1 and RB may impact or regulate BLM or could be a common target of tumor suppressors.

The final pertinent question will be whether other mechanisms exist in cancer cells that allow BLM to act as an oncogene under certain conditions during cancer progression, whether these mechanisms act in conjunction with each other, or whether there are any specific networks that aid BLM to act as a double-edged sword. Exploration studies on these lines will allow a better understanding of the rewiring of BLM, and its Janus-like character may have important implications in the study of neoplastic transformation and cancer development.

## Author Contributions

SS conceptualized the review and gave overall inputs. EK and RA wrote the drafts and generated the figures. EK, RA, and SS together generated the final version. All authors contributed to the article and approved the submitted version.

## Conflict of Interest

The authors declare that the research was conducted in the absence of any commercial or financial relationships that could be construed as a potential conflict of interest.
